# Beyond the youth success trap: a Four-Gate Coaching Audit of Japan's 2017 U20 football cohort

**DOI:** 10.3389/fspor.2026.1894940

**Published:** 2026-08-21

**Authors:** Rui Chen, Shiyan Sheng, Xiaoyu Liu

**Affiliations:** School of Football , Wuhan Sports University, Wuhan, China

**Keywords:** elite youth football, youth-to-senior transition, talent identification, audit protocol, longitudinal monitoring, FIFA U-20 World Cup

## Abstract

**Introduction:**

Youth tournament success is visible, but it is not itself evidence of later adult access, elite club exposure or senior national-team contribution. Coaching departments therefore require source-traceable follow-up after selection. This methodological case study introduces a proof-of-concept Four-Gate Coaching Audit for converting a youth selection event into a longitudinal, player-level evidence register.

**Methods:**

The audit is a monitoring protocol rather than a validated prediction model. It separates four non-substitutable constructs: documented selection context, post-youth adult competitive access, club-only Post-Youth Elite Exposure (PEEX) and Senior Contribution Ladder (SCL) status. Codes are assigned only from official federation, competition, league or club records; unestablished evidence is coded as NE rather than zero. The demonstration uses the complete 21-player final participant cohort of Japan at the FIFA U-20 World Cup Korea Republic 2017.

**Results:**

Tournament-time foreign-club exposure was confirmed for 1/21 players (4.8%), whereas post-youth adult competitive access was officially verified for all 21 players. Under the primary strict senior-tournament rule, SCL-3/4 was confirmed for at least 6/21 players (28.6%); under a broader rule including played Copa América 2019 minutes, this increased to 9/21 (42.9%). Club-only PEEX-3/4 was confirmed for at least 10/21 players (47.6%) after official-source reconciliation. Raw intercoder agreement was high, but source reconciliation still corrected evidence-sensitive classifications, supporting the need to distinguish reliability from endpoint verification.

**Discussion:**

The study contributes a construct-separating audit protocol for monitoring youth-to-senior pathways without treating youth selection, overseas registration or senior-team association as automatic evidence of conversion. The findings are official-confirmed minimums and do not imply causal effects, player-quality rankings or national-system superiority.

## 1 Introduction

Success in youth international football is visible, memorable, and institutionally useful; however, its meaning is easily overstated. A youth result records what a selected group achieved at an age-grade tournament; it does not establish whether individual players later gained adult competitive access, entered high-exposure club settings, or contributed in senior international competition. The coaching problem therefore begins after selection: How can staff follow a cohort without turning a roster label into a premature conversion claim?

This manuscript introduces the Four-Gate Coaching Audit as a proof-of-concept solution to that problem. Its novelty lies not in a new theory that predicts elite careers but in an evidence architecture that separates four commonly conflated claims: documented selection context, postyouth adult competitive access, club-only elite exposure, and senior national-team contribution. The audit was developed in response to an applied monitoring need in coaching departments: after a youth tournament, staff require a defensible record of what happened next and which official source supports each claim.

Three literature works justify this approach. Football-specific and broader elite-sport studies show that youth international selection and junior success are limited and imperfect predictors of senior attainment (1–8). Transition research shows that adult opportunity, role continuity, organisational alignment, loan or transfer decisions, setback management, and environmental fit shape whether selected youth players can move into meaningful senior contexts (9–17). Migration and measurement validity work further cautions that overseas registration, senior association, and public reputation should not be interpreted as outcomes unless the evidence source matches the claim being made (18–20).

The operational gap is therefore not whether youth-to-senior transitions are uncertain; that fact is well established. Instead, the challenge lies in how a technical department can convert a single youth selection event into a transparent follow-up record. The audit freezes the final participant cohort and then asks four sequential questions: Who was selected and in what context? Who later gained adult competitive access? Who reached a defined club-exposure band? Who reached recurrent or event-level senior national-team involvement?

Japan's 2017 U20 cohort serves as a diagnostic case rather than a representative national model. It is analytically useful because the official youth roster shows very low tournament-time foreign-club exposure, whereas later official records show adult access, club-only high-exposure evidence, and senior integration for different subsets of players. Therefore, the case tests whether the audit prevents a simple overseas-at-youth-tournament narrative from substituting for later pathway evidence.

The study asks three questions: (1) Can all four audit gates be populated conservatively from official public evidence for one complete youth-tournament cohort? (2) Does tournament-time foreign-club exposure under-describe later officially confirmed club-only Post-Youth Elite Exposure (PEEX)-3/4 exposure? (3) How sensitive are senior-integration counts to broad versus strict definitions of eligible senior final-tournament deployment?

This paper therefore makes three linked contributions. First, it reframes youth-tournament achievement as the starting point for pathway accountability rather than as a conversion verdict. Second, it specifies a construct-evidence match, establishing that selection, adult access, club exposure, and senior deployment are distinct claims requiring distinct official records. Third, it treats NE as non-establishment, so missing or insufficient public evidence is not silently converted into a negative outcome. These contributions position the audit as a measurement-validity and decision-support protocol rather than a causal model of national talent development.

## 2 From selection outcome to pathway accountability

Public discussion often treats a youth tournament result as an analytic endpoint, whereas transition research points in the opposite direction: the relevant unit is the pathway through opportunities, environments, and selection decisions that follow youth identification. Academy and first-team transition studies identify adult opportunity, role fit, support structures, and organisational timing as conditions that shape whether youth promise translates into senior participation (9–17). A pathway audit therefore needs to begin where the public narrative often stops.

The proposed audit frames postselection monitoring as a form of pathway accountability. A youth roster establishes a denominator and a selection context, but it cannot show whether a player gained adult access, entered a high-exposure club environment, or contributed to a salient senior tournament. Each later claim must be supported by evidence that is specific to that claim rather than inferred from the original selection event.

The audit applies three rules. First, the denominator is fixed at the final participating cohort so that replacements and injury departures do not distort follow-up. Second, the evidence type must match the construct: roster evidence supports selection context, league or club evidence supports adult and club exposure, and federation match evidence supports senior deployment. Third, insufficient public evidence is coded as NE rather than as absence. These rules are the methodological core of the Four-Gate Coaching Audit.

The official primary rule follows from this construct-evidence logic. Official records reduce the risk that media narratives, aggregator drift, or retrospective reputation will determine endpoint classifications. While they do not eliminate bias—public official archives may be incomplete, uneven across competitions, or less accessible for lower-profile contexts—the audit addresses this by reporting officially confirmed minimums and preserving NE as a documentation boundary.

## 3 Four-Gate Coaching Audit

**Figure 1:**
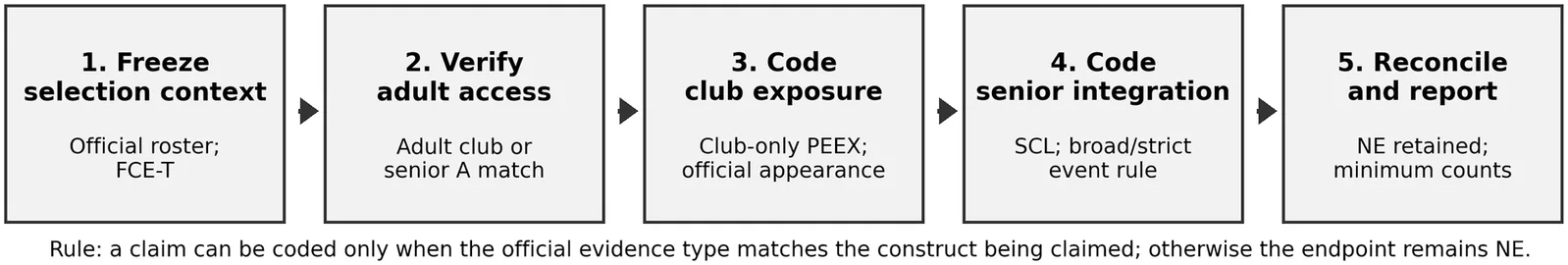
Evidence flow for the official-primary Four-Gate Coaching Audit. FCE-T, foreign-club exposure at tournament date; PEEX, postyouth elite exposure; SCL, senior contribution ladder; NE, not established in retrieved official evidence.

The framework is a monitoring protocol rather than a deterministic theory of progression. Gate 1 records documented selection context, Gate 2 verifies postyouth adult competitive access, Gate 3 assigns club-only PEEX as an ordinal exposure flag, and Gate 4 assigns Senior Contribution Ladder (SCL) as an ordinal senior-integration salience flag. The four gates deliberately separate constructs that are often conflated in narrative accounts of talent conversion. The evidence flow and operational evidence rules are shown in Figure 1 and Table 1.

**Table 1:** Operational audit framework and evidence rule.

Gate	Measured construct	Coded evidence in this study	Guardrail
1. Selection context	Roster denominator and FCE-T	JFA final participant roster and change notice	Selection is documented; validity is not inferred
2. Adult access	Postyouth adult playing access	Official club/league match or senior A-match evidence	Verified minimum; NE is not absent
3. High-exposure club pathway	Exposure-status band of the club pathway	Official club/league first-team evidence; club-only PEEX	Ordinal exposure flag, not a universal ranking
4. Senior integration	Salient senior participation	Official A-international records and played final-tournament minutes	SCL salience is reported with caps where evidenced

All four gates receive a row-level status in this cohort. Codes not established from retrieved official evidence are considered NE rather than zero.

## 4 Methods

### 4.1 Design, cohort, and case-selection rationale

The study employs a methodological case study design (21) centred on a sole empirical cohort: Japan at the FIFA U-20 World Cup Korea Republic 2017. The official JFA squad record lists 21 selected players and notes that Akito Takagi replaced the injured Tsukasa Morishima on 12 May 2017, establishing an audit denominator of 21 final participating players. Although the record also notes Koki Ogawa's subsequent injury departure, he remains part of the tournament participant cohort (22).

The case was selected for three reasons. First, the official roster provides a clear, complete denominator, enabling a closed-cohort audit rather than a selective list of successful players. Second, the cohort presents a diagnostic contrast between very low tournament-time foreign-club exposure and later visible adult pathways, which directly tests the danger of relying on FCE-T as a pathway proxy. Third, sufficient official public records were retrievable to populate adult access, PEEX, and SCL endpoints at the player level. These features make the case well suited for demonstrating the protocol rather than for generalising about all Japanese cohorts.

The value of the single-cohort design lies in auditability rather than representativeness. A closed official roster ensures that every selected player remains in the denominator, every endpoint is traced to a specified evidence type, and every non-established claim remains visible as NE. This makes the case suitable for demonstrating whether a youth selection event can be converted into a longitudinal, source-traceable register before the protocol is extended to comparative or explanatory designs.

No six-cohort comparative baseline is analysed. A valid cross-cohort analysis would require equivalent, player-specific official evidence for roster identity, adult access, exposure, and senior endpoints across all cohorts. Such evidence was not fully recoverable on a comparable basis for all previously contemplated cohorts. Restricting inference to a single complete official-roster cohort is therefore methodological rather than merely practical: it is preferable to an apparently broader comparison that relies on non-equivalent or partially traceable evidence.

### 4.2 Source hierarchy and traceability

Assignments were made only from official public records accessed on 27 and 28 May 2026 (22–29). Tier 1 comprises the JFA roster, match sheet, and official programme evidence. Tier 2 comprises official competition or league records, including those of the J.LEAGUE, Bundesliga, Premier League, and LaLiga. Tier 3 comprises an official club report when it explicitly documents competition appearances. Public aggregator databases, media reports, and transfer-market pages were not used to assign an outcome code.

Supplementary File 1 records the exact official URL, source type, access date, and coding note for each player-level supporting record. Match sheets and maintained player profiles are preferred when available because they offer greater stability and repeatability as endpoint sources. Official news or club reports are retained only when they explicitly state the qualifying appearance fact, ensuring their less structured source type remains visible to the reviewer. The audit thus distinguishes official provenance from documentary stability.

Because the audit uses named player rows, an ethical check was added to the source protocol. This paper reports only information already available in official federation, league, competition, or club records and uses player names as public sport identifiers required for audit traceability. No private interviews, medical information, performance testing, or unpublished personal data were collected. Injury information is reported only where it forms part of the official tournament roster record. The use of names is therefore limited to reproducible public-record verification; the audit should anonymise or aggregate rows when applied to non-public youth, academy, or school settings. The endpoint-specific official-source hierarchy is summarised in Table 2.

**Table 2:** Evidence hierarchy for the official-primary audit.

Endpoint	Eligible evidence	Use
Cohort/FCE-T	Official JFA 2017 U20 roster and changes	Complete the denominator and tournament-time club association
SCL-4 strict	JFA World Cup and AFC Asian Cup match sheets	Playing minutes in Japan's confederation/FIFA final tournaments
SCL-4 broad	Strict evidence plus JFA Copa America match sheets	Sensitivity specification acknowledging invited tournament status
SCL-3	Official dated senior cap record	At least 10 A-international appearances; dated value retained
PEEX	Official league or club first-team appearance record	Club-only exposure; no SCL event route reused

Supplementary File 1 records exact player-level URLs and source types. Supplementary File 2 contains the two raw coder files: the paired comparison log and the reconciliation log.

### 4.3 Coding of the four gates

FCE-T equals Yes when a player's tournament-time club association differs from the represented association in the official youth roster. Gate 2 equals Verified when an official record confirms at least one postyouth adult club-competition appearance or senior A-international playing appearance. If the retrieved official record set fails to establish the endpoint, the code is recorded as NE.

SCL is an integration-salience flag rather than a better-player scale. SCL-3 requires an official dated record of at least 10 senior A-international appearances. SCL-4 requires actual playing participation in an eligible senior final tournament. Under broad SCL-4, Japan's matches played in the Copa América 2019 are eligible because the nation officially participated as a senior team. Under strict SCL-4, only playing minutes in the FIFA World Cup and AFC Asian Cup are eligible. Strict SCL serves as the primary, event-sensitive interpretation.

PEEX is an ordinal coaching exposure-status flag rather than a universal league-strength ranking. PEEX-1 records repeated domestic professional access through at least 10 official J1 appearances. PEEX-2 records Asian continental club participation. PEEX-3 records verified European first-team exposure outside a Big Five top-tier or in a European second professional tier. PEEX-4 records verified Big Five top-division first-team exposure. The main high-exposure summary is club-only PEEX-3/4, so it cannot be mechanically produced by senior-tournament evidence. Because these thresholds are contestable by design, logic and boundary checks for them are specified in the following section.

### 4.4 Threshold logic, conservative cut points, and sensitivity checks

The PEEX and SCL thresholds were selected based on four design criteria rather than derived as universal cut scores: they had to be recoverable from official public records for all cohort members, interpretable for coaching staff, conservative against symbolic or de minimis participation, and open to sensitivity testing. They are therefore monitoring categories, not rankings of player quality, league strength, or developmental success. This design draws on transition literature concerning sustained adult opportunity and environmental fit (7–17), migration and measurement validity cautions regarding the over-reading registration labels (18–20), and match-analysis research that commonly treats the English Premier League, Bundesliga, La Liga, Serie A, and Ligue 1 as the “Big Five” European leagues while acknowledging between-league differences (30, 31).

For PEEX-1, at least 10 J1 appearances were used to mark repeated domestic first-team opportunities. A single debut, a short substitute episode, or a cup listing would be too fragile as evidence that adult access has become established. A five-appearance rule would improve sensitivity but risk over-classifying brief exposure; a 20-appearance rule would be stricter but could exclude players who had already crossed a meaningful repeated-access boundary. Because the main high-exposure endpoint is club-only PEEX-3/4, this domestic threshold is reported with a 5/10/20 sensitivity check rather than treated as a decisive conclusion.

PEEX-3 and PEEX-4 distinguish domestic repeated access from officially evidenced cross-context club exposure. PEEX-3 flags first-team participation in a European top-flight outside the Big Five or in a European second professional tier. PEEX-4 is reserved for Big Five top-division first-team participation because the coded construct represents access to a globally prominent club-competition setting. These categories deliberately do not claim that Europe is a universal developmental destination, that every European pathway is superior to every non-European pathway, or that a single appearance equates to a sustained contribution.

For SCL, the 10-cap threshold for SCL-3 separates recurrent senior national-team involvement from symbolic selection or a one-off appearance. SCL-4 requires actual playing minutes in an eligible senior final tournament because the construct represents event-level deployment rather than squad association. The broad specification includes playing minutes from the Copa América 2019 because Japan officially participated in the event, whereas the strict specification excludes the Copa América 2019 because Japan competed as an invited guest rather than through the ordinary CONMEBOL confederation qualification route.

These boundary decisions are reported as analytic assumptions rather than background details. The J1 appearance sensitivity check tests whether domestic repeated-access counts depend on the chosen threshold. The broad/strict SCL comparison tests whether senior-integration counts depend on treating Copa América 2019 as equivalent to Japan's FIFA/AFC final-tournament route. The operational rules, rationale, and boundary protections are summarised in Table 3.

**Table 3:** Threshold logic, rationale, and sensitivity protection for PEEX and SCL.

Indicator	Operational rule	Rationale and alternatives considered	Boundary protection
PEEX-1	≥10 official J1 appearances	Marks repeated domestic first-team opportunity. Five appearances would be more sensitive but may over-classify brief access; 20 would be stricter but may miss meaningful repeated access	Reported with 5/10/20 sensitivity checks; does not determine the main PEEX-3/4 high-exposure endpoint
PEEX-3	European top flight outside the Big Five or the European second professional tier	Flags official cross-context European first-team exposure that is distinct from domestic J1 repetition	Exposure flag only; not a claim that all European pathways outrank all non-European pathways
PEEX-4	Big Five top-division first-team appearance	Flags access to a globally prominent comparative club setting used in published football match-analysis literature (30, 31)	Not a minutes-weighted contribution, quality ranking, salary proxy, or universal league-strength claim
SCL-3	≥10 senior A appearances	Separates recurrent senior involvement from symbolic selection or one-off appearance	Operational monitoring cut point only; future studies can test alternative cap thresholds using complete official data
SCL-4	Played senior final-tournament minutes	Captures event-level deployment rather than squad association; broad and strict rules address Copa América 2019	Broad rule includes minutes played in the Copa América 2019; the strict rule limits the endpoint to FIFA World Cup and AFC Asian Cup minutes

PEEX and SCL are conservative monitoring flags rather than validated performance scales. PEEX records the highest officially recorded club-exposure status achieved under the stated rules; it does not rank players, minutes, salaries, market value, or global league quality. SCL records recurrent senior involvement or eligible final-tournament deployment; it does not rank player value or national-team importance.

### 4.5 Analysis and intercoder reliability

The analysis is descriptive. Counts and percentages use the fixed denominator of 21, and endpoints with NE rows are reported as official-confirmed minimums. No inferential test or causal comparison is appropriate for this diagnostic demonstration.

Two coders independently classified the 21 endpoint rows using the same codebook, endpoint definitions, and official-primary source hierarchy. The second coder completed the workbook before reconciliation and before any revisions to the endpoint counts. The full workflow, raw paired assignments, and coding fields are reported in Supplementary File 2.

Percentage agreement and Cohen's kappa were calculated on the submitted raw assignments where variation permitted estimation. Disagreements and concordant cases requiring source correction were then adjudicated through official-source reconciliation. Only the essential reliability summary is retained in the main Results section; detailed statistics and reconciliation notes are in Supplementary File 2.

## 5 Results

### 5.1 Selection context and adult access

Unless explicitly described as complete, all percentages in the results represent official-confirmed minimums. NE indicates that an endpoint was not established from the retrieved official evidence, rather than implying that the outcome did not occur.

The JFA roster and player-change notice fix the final participant denominator at 21. Only Louis Yamaguchi was registered with a non-Japanese club at the time of the tournament, and official evidence verified subsequent adult competitive access for every player. The endpoint counts are summarised in Table 4.

**Table 4:** Official-confirmed endpoint summary, Japan 2017 U20 cohort (*n* = 21).

Endpoint	Count	Percentage	Interpretation
FCE-T Yes	1/21	4.8%	Youth tournament-time overseas registration was rare
Adult access verified	21/21	100.0%	All players had official adult-access evidence
Strict SCL-3/4	6/21	28.6%	Conservative senior-integration minimum under FIFA/AFC final-tournament rules plus SCL-3 caps
Broad SCL-3/4	9/21	42.9%	Copa América 2019 inclusion changes the senior-integration classification
Club-only PEEX-3/4	10/21	47.6%	High club-exposure status substantially exceeds the FCE-T signal

FCE-T, foreign-club exposure at tournament date; SCL-3/4 = official-confirmed senior-integration status under the stated rules; PEEX-3/4 = official-confirmed club-only high-exposure status. Full player-level codes, URLs and source notes are reported in Supplementary File 1.

### 5.2 Official endpoint summary and player-level register

Table 4 summarises the main official-confirmed endpoint counts. The complete player-level register, including URLs and access dates, is provided in Supplementary File 1 so that the main text does not duplicate row-level tabular evidence.

### 5.3 Worked examples of row-level coding

Table 5 presents three contrasting rows to show how the audit prevents construct substitution. Detailed URLs and access dates are provided in Supplementary File 1.

**Table 5:** Worked examples from official evidence to audit code.

Player	Official evidence trail	Assigned codes	Interpretation
Takefusa Kubo	JFA U20 roster; JFA World Cup/AFC Asian Cup match records; official LaLiga first-team record	FCE-T No; Access V; Strict S4; Club P4	Domestic registration at U20 coexists with later evidence from strict senior and Big Five club
Koji Miyoshi	JFA U20 roster; JFA Copa América playing record; official Bundesliga match record documenting playing participation and a goal	FCE-T No; Access V; Broad S4; Strict NE; Club P4	Copa sensitivity changes SCL, while a separate official Bundesliga record establishes Big Five club exposure
Louis Yamaguchi	JFA roster lists FC Lorient/France; official J.LEAGUE profile documents adult access and at least 10 J1 appearances	FCE-T Yes; Access V; SCL NE; Club P1	Overseas tournament-time status does not automatically establish later high exposure or senior integration

As shown in Table 5, the same player can carry different evidence statuses across gates; this is why FCE-T, PEEX, and SCL are not treated as interchangeable outcomes.

### 5.4 Senior integration and event sensitivity

**Figure 2:**
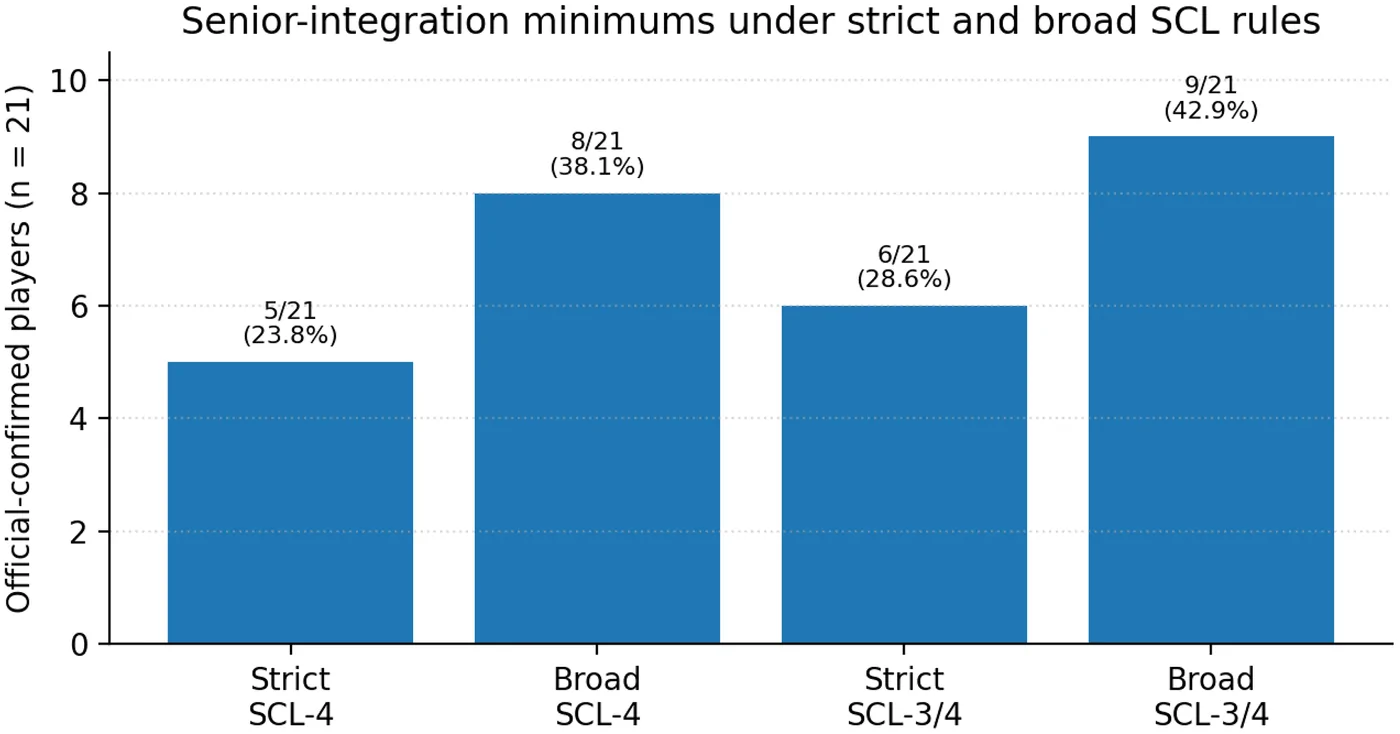
Official-confirmed senior-integration minimums under strict and broad SCL specifications, Japan 2017 U20 cohort (*n* = 21). Strict SCL-4 includes only playing minutes in the FIFA World Cup and AFC Asian Cup; broad SCL-4 additionally includes playing minutes in the Copa América 2019. SCL-3 captures official evidence of at least 10 senior A-international appearances.

Because SCL-4 depends on the eligible-event rule, strict and broad specifications are reported side by side (Figure 2). Official records also confirm recurrent senior involvement for players meeting the SCL-3 threshold. One reconciled correction was retained for Ryosuke Kojima because the official J.LEAGUE profile reports zero Japan senior appearances.

The count pattern in Figure 2 should therefore be read as an event-definition sensitivity result rather than as a single conversion score. Player-level identities and source trails for the strict and broad specifications are provided in Supplementary File 1.

### 5.5 Club-only high-exposure club status and evidence status

The primary club-exposure conclusion concerns club-only PEEX-3/4, while domestic PEEX-1 threshold checks are treated as boundary tests. After integrating both submitted coding records and official-source reconciliation, official club or league evidence confirms club-only PEEX-3/4 for at least 10 of 21 players. Evidence-status categories for the reported endpoints are presented in Table 6.

**Table 6:** Evidence-status summary for reported endpoints.

Endpoint	Positive official evidence	Explicit official zero	NE/no positive assignment	Interpretation
FCE-T Yes	1	20	0	Complete roster-based classification
Adult access	21	0	0	Complete official verification
Senior A appearance	12	6	3	Confirmed minimum; three rows lack explicit official senior status evidence
Broad SCL-4	8	0	13	Includes played Copa América evidence
Strict SCL-4	5	0	16	Primary final-tournament endpoint; WC/AFC Asian Cup minutes only
Club-only PEEX-3/4	10	0	11	Adjudicated official-confirmed minimum independent of senior tournament coding

FCE-T and adult access are complete classifications in this cohort; remaining positive endpoint counts are interpreted under the aforementioned official-confirmed minimum rule.

Table 6 and Supplementary Files 1–S2 provide the detailed status and reconciliation trail. The main interpretation is limited: PEEX-3/4 is an official-confirmed exposure minimum, and NE remains a non-establishment code rather than a verified negative outcome.

### 5.6 Intercoder agreement and source reconciliation

Because intercoder reliability was not the primary empirical outcome, only the essential summary is reported here, while full paired assignments, category counts, kappa values, and reconciliation notes are provided in Supplementary File 2. Agreement reached 21 out of 21 for Gate 2, broad SCL, and strict SCL, and 19 out of 21 for club-only PEEX. Source reconciliation corrected one concordant broad-SCL assignment for Ryosuke Kojima and accepted two PEEX revisions for Go Hatano and Teruki Hara after retrieving qualifying official evidence. Reliability therefore supports reproducibility, but final endpoint reporting still depends on source-traceable verification.

## 6 Discussion

### 6.1 Principal findings and their interpretation

The central finding centres on construct non-equivalence rather than a single conversion rate. The construct is represented by four distinct endpoints: tournament-time foreign-club exposure (1/21), later adult competitive access (21/21), club-only PEEX-3/4 (at least 10/21), and two SCL specifications. Each gate addresses a different question using a distinct follow-up window and evidence requirement. The reconciliation results reinforce this point. High raw intercoder agreement did not prevent one shared SCL correction and two PEEX revisions, indicating that reproducible coding cannot substitute for endpoint verification. The audit's main contribution is therefore to prevent selection context, adult access, club exposure, and senior deployment from being treated as interchangeable evidence of pathway success.

The 21/21 adult-access finding requires particularly careful interpretation. Although it may appear more favourable than prior transition research, the endpoints and denominators are not equivalent. Dugdale et al. followed 537 academy players and found that only 53 obtained a professional contract at the study club; recruitment at a younger age did not increase that probability (8). Herrebrøden and Bjørndal likewise showed that youth international experience was only a limited predictor of later senior elite participation across nations and positions (1). The present cohort had already passed a stringent national-team selection filter, and Gate 2 records only verified adult competitive access rather than sustained minutes, league level, or career stability. Universal adult access therefore does not contradict the lower transition rates reported elsewhere, nor does it show that youth selection reliably predicts an established elite career.

### 6.2 Comparison with previous research and plausible influences on the observed pattern

The divergence between tournament-time affiliation and later PEEX status is consistent with transition research that treats progression as a changing interaction between the player and the developmental environment. Swainston et al. followed players prospectively and found that adaptation to senior football involved greater physical and mental demands, changes in playing style, and pressure to win. Limited first-team opportunity was also associated with subsequent loans and changes in club identity, motivation, and confidence (9). McGuigan et al. identified a lack of opportunity and a lack of development-specific coaching as transition barriers, alongside technical, physical, and environmental influences (15). These findings suggest that accumulated opportunity, role fit, coaching decisions and adaptation across several seasons may shape later exposure. Although the current audit cannot test those mechanisms, it identifies the players and time points for which such explanations should be investigated.

Organisational continuity offers a second plausible influence. Relvas et al. found that limited proximity and formal communication between youth and professional environments appeared to hinder coherent player progression, despite broadly similar formal academy structures across European clubs (11). A recent scoping review characterised the junior-to-senior transition as a multifactorial process shaped by psychosocial, organisational, and physical factors rather than by a single selection event (32). In the Irish player pathway, Sweeney et al. reported weak horizontal and vertical stakeholder coherence, including inconsistent understandings of long-term pathway aims (33). Although these studies are contextual rather than direct comparators for the Japanese cohort, they expose what the four gates fail to measure. PEEX status records verified exposure rather than the communication, support, training load, injury history, or sequence of decisions that produced it.

The FCE-T and PEEX contrast also qualifies migration-based interpretations. Research on the Bosman ruling demonstrates that regulation can alter football labour mobility (18), while cross-national work links player migration and international performance at an aggregate level (19). These studies provide background on mobility but do not test individual youth-to-senior pathways. In this cohort, FCE-T is a one-date descriptor, whereas PEEX uses longitudinal first-team evidence. The contrast between 1/21 at FCE-T and at least 10/21 at PEEX-3/4 may therefore reflect the timing of observation and subsequent mobility as much as any developmental advantage. Domestic registration at the U20 tournament should not be treated as limited potential, just as overseas registration should not be treated as proof of successful progression.

The strict and broad SCL results point to a measurement issue rather than a pathway mechanism. The player histories remain unchanged, while the count varies depending on the definition of an eligible senior event. This distinction accords with the principle that an operational indicator must remain aligned with the intended construct (20). Reporting the strict specification as the primary endpoint and the broad specification as a sensitivity analysis makes the event-equivalence assumption visible. It does not imply that the underlying level of senior contribution changed when the Copa América 2019 was included.

### 6.3 Alternative explanations and interpretive boundaries

The observed pattern has at least two plausible explanations that do not require an unusually effective transition pathway. First, the 21/21 adult-access count may partly reflect prior selection into a national youth team and the permissive Gate 2 threshold of any verified adult appearance. Second, the gap between FCE-T and later PEEX may reflect the timing of observation and subsequent labour mobility rather than a developmental effect of club environment. The design cannot separate these explanations because it contains no unselected comparator group and no direct measures of opportunity, role stability, training, or support.

Official-primary coding introduces a different source of uncertainty. Historical records, lower-profile competitions, and federation archives may be incomplete, uneven, or inaccessible. Consequently, NE is an evidence boundary rather than a verified negative outcome, and the probability of NE may differ across players, competitions, and countries. PEEX and SCL counts should therefore be interpreted as officially confirmed minimum counts. Missing evidence could depress the observed exposure counts, whereas the permissive adult-access threshold could inflate the apparent breadth of progression relative to more demanding transition definitions.

These boundaries limit the claims that can be made from the case. A single, highly selected cohort cannot estimate a population transition probability, describe the pathways of unselected players, or isolate effects of the Japanese development system. The PEEX and SCL bands are operational monitoring categories rather than validated rankings of player quality, league strength, salary, tactical contribution, or well-being. Although the coding exercise showed high raw agreement, the source corrections indicate that further independent applications are required before the protocol can be considered validated across settings. The results should not be combined into a single success score or used for causal explanation, benchmarking, or claims of national-system superiority.

### 6.4 Mitigation and applied implementation

Some of these limitations can be mitigated, although none of the proposed procedures can turn this design into a causal study. Future applications should preregister the cohort denominator, follow-up window, eligible competitions, evidence hierarchy, and threshold alternatives before coding begins. Threshold sensitivity should be reported alongside the primary specification. Official records should be triangulated across federation, competition, league, and club sources, with archived copies and access dates retained. Independent recoding and documented reconciliation should then be repeated at scheduled updates. These procedures cannot remove missing information or selection effects, but they make the direction and location of uncertainty visible.

For technical departments, the four gate codes should remain concise outcome fields, while a linked process log records the context needed for action. Useful fields include competitive minutes, positional role, loan or transfer episodes, injury-related availability, training exposure, and the staff member responsible for follow-up. This separation prevents contextual information from silently changing a gate definition. It also addresses an organisational problem identified in previous research. Gregson et al. found substantial variation in academy staffing and data practices, with medical and performance data integrated in the same management system more often in clubs than in national federations (34). A shared, source-tracked register may therefore support communication, but its implementation must align with local staffing and information infrastructure.

The implementation schedule and hypothetical cases are included in Appendix A to keep the Discussion focused on interpretation. They are design propositions that illustrate how the audit could be used; they are not evaluated interventions or evidence that maintaining the register improves decisions.

### 6.5 New questions and future research

The next research question is not simply how many youth internationals converted, but which evidence-defined pathway states emerge, what processes precede them, and how consistently they can be measured. The first validation step should apply unchanged rules to another Japanese age-grade cohort. This would test within-system replicability while limiting variation in federation and league records. A second study should examine another Asian cohort and preregister equivalent and alternative thresholds. Cross-system studies should follow only after the evidence rules can be implemented consistently.

Future research should test whether the audit has explanatory and decision-making value without reducing it to a deterministic prediction score. Longitudinal studies could examine whether minutes, role continuity, loans, injuries, stakeholder communication, and support explain movement between gates. Comparative work could then examine reserve-team, export, and collegiate pathways across regions, while explicitly testing measurement equivalence and differences in archive completeness. This staged sequence preserves construct separation and avoids attributing cross-system differences to player development when they may arise from non-equivalent records or competition structures. Future work should also involve structured input from coaches, technical directors, and academy staff in refining the gate definitions, and should compare the Four-Gate Audit against existing youth-to-senior transition or talent-monitoring frameworks to establish its relative value.

A final question concerns applied impact. It remains unknown whether maintaining the audit changes coaching decisions, improves communication, or helps departments identify support needs earlier. Prospective implementation studies should therefore compare decision processes before and after adoption, document how practitioners use NE cases, and assess whether scheduled reviews lead to timely action. Such evidence would move the Four-Gate Coaching Audit from a transparent proof of concept towards a validated monitoring practice, while retaining the boundaries demonstrated by this single-cohort case.

## 7 Conclusion

This article introduces, rather than validates, a Four-Gate Coaching Audit for turning a youth roster into a source-traceable pathway register. In Japan's 2017 U20 cohort, 1/21 players had tournament-time foreign-club exposure, whereas at least 10/21 later met club-only PEEX-3/4. This contrast illustrates why selection context and longitudinal exposure should not be treated as the same outcome. The adult-access, SCL-sensitivity, and reconciliation findings further show that pathway conclusions depend on the specified construct, threshold, and evidence source.

The practical contribution is a disciplined monitoring logic for coaching departments. A youth result becomes the start of follow-up rather than the end of interpretation. Endpoint claims remain linked to official sources, while endpoints that cannot be established remain visible as NE. Although the protocol may strengthen pathway accountability, its explanatory value, cross-system validity, and effect on coaching decisions require prospective evaluation beyond this single cohort.

## 8 Practical applications

Technical directors and coaching staff can use the audit to prevent premature judgments about conversion based on a youth tournament result, an overseas registration label, or a senior call-up. The protocol requires the corresponding evidence type for each claim: adult match access, club-only high-exposure status, or a contribution to a senior tournament. Reporting strict and broad SCL specifications also prevents Japan's invited Copa América 2019 participation from being automatically treated as equivalent to World Cup or AFC Asian Cup deployment.

## Data Availability

The datasets generated and/or analysed for this study are included in the Supplementary Material.

## Appendix A. Illustrative implementation framework

This appendix retains the practice-oriented schedule and hypothetical cases previously presented in the Discussion section. They illustrate a possible implementation sequence and do not constitute evaluated outcomes or recommendations supported by an intervention study.

**Table A1:** Illustrative implementation schedule for technical departments.

Timing	Technical department action	Official evidence source	Warning signal	Possible response
Within 1 month of the youth tournament	Freeze roster denominator, role, and FCE-T	Federation roster/change notice	Roster or club status incomplete	Create a source-tracked cohort register
Post-tournament year 1	Verify adult competitive access	League/club official match records	No established adult match evidence	Review the pathway plan and club communication
Years 2-3	Update club-only PEEX and evidence status	Official league, club, and continental records	Registration without verified playing exposure	Review loan/platform fit and opportunity
Years 5–8	Update SCL and event sensitivity	Federation squads, programmes and match sheets	Call-up without verified minutes	Analyse position competition and integration support
Annually	Revisit NE rows and commission recoding	Updated official records; independent coder log	Persistent NE or coding disagreement	Report the evidence gap and reconcile transparently

This schedule is a decision-support application of the audit rather than an evaluated intervention.

A prospective example illustrates the distinction between recording and intervention. Suppose a 21-player U20 cohort enters the register in 2027. In year 1, Player A remains NE for adult access because no official match evidence is available. Staff should first verify the evidence trail, then review opportunity and club communication without assigning a failure label. In year 3, Player B signs for a European club but records no verified first-team appearance. The move is logged as context, while PEEX-3/4 remains unassigned, and the pathway review considers playing opportunity, role, and loan options. In year 5, Player C reaches 12 senior caps without eligible final-tournament minutes. SCL-3 is assigned, SCL-4 is withheld, and the audit records a specific integration state rather than an overall judgement. By year 8, the register separates verified outcomes, unresolved evidence, and support questions that require practitioner interpretation. These cases are hypothetical and should not be interpreted as observed effects of the audit.
